# Osteocytic osteolysis: time for a second look?

**DOI:** 10.1038/bonekey.2012.229

**Published:** 2012-12-05

**Authors:** John J Wysolmerski

**Affiliations:** 1 Department of Internal Medicine, Section of Endocrinology and Metabolism, Yale University School of Medicine, New Haven, CT, USA

## Abstract

Over 100 years ago it was suggested that osteocytes could remodel their surrounding environment by removing and replacing bone. In the 1960s and 1970s, many observations were made to suggest that osteocytes could resorb bone and increase the size of their lacunae. This concept became known as osteocytic osteolysis and studies suggested that it occurred in response to diverse stimuli such as parathyroid hormone, calcium restriction, hibernation and reproductive cycles. However, this concept fell out of favor in the late 1970s when it became clear that osteoclasts were the principal bone-resorbing cells in the skeleton. Over the past decade, we have increasingly appreciated that osteocytes are remarkably versatile cells and are involved in all aspects of skeletal biology, including the response to loading, the regulation of bone turnover and the control of mineral metabolism. Recent data have demonstrated that osteocytes remodel their perilacunar and canalicular matrix and participate in the liberation of skeletal calcium stores during lactation. In light of these new findings, it may be time to reassess the concept of osteocytic osteolysis and reconsider whether osteocyte lacunar and canalicular remodeling contributes more broadly to the maintenance of skeletal and mineral homeostasis.

## Introduction

Over the last decade bone biologists and endocrinologists have increasingly appreciated the versatility of osteocytes as new evidence has underscored the involvement of these cells in regulating surface bone cell activity and mineral metabolism.^1^,^2^,^3^,^4^,^5^ Osteocytes are characterized by their cell lineage, their typical location and their extensive interconnectedness. They arise from mesenchymal stem cells and are derived from the osteoblast lineage.^1^,^2^ Through an active signaling process that is only partly understood, some osteoblasts decide to become osteocytes and send out cellular projections into osteoid, make contact with the established osteocyte network and become surrounded by mineralized bone.^6^,^7^,^8^,^9^,^10^ The most obvious features of these cells are the extensive dendritic processes that extend from each cell and make contact with other osteocytes.^1^ The presence of gap junctions at the borders between processes from different cells enables osteocytes to form a functional network, which also communicates with surface bone cells at both the periosteum and endosteum.^1^,^2^,^11^ Osteocytes and their dendritic processes reside within a series of interconnected lacunae and canaliculi within the mineralized bone tissue that are in close communication with the vascular space. As a result, the lacunar-canalicular network represents an enormous area of contact between bone mineral and the extracellular fluid, one that is much larger than the periosteal, endosteal and trabecular surfaces combined.^1^,^12^ The osteocyte network has been shown to translate the effects of mechanical force into bone cell activity and the regulation of bone mass.^1^,^2^,^13^ It has been increasingly recognized as important in orchestrating bone turnover.^1^,^2^,^14^,^15^,^16^,^17^ Osteocytes also are central to the regulation of systemic phosphate homeostasis, and coordinate bone mineralization with FGF23 production and renal phosphate handling.^1^,^4^,^5^ However, the enormous surface area of the osteocyte canalicular network potentially makes these cells an ideal site for calcium and phosphorus exchange between the circulation and the skeleton. That osteocytes can remove and deposit bone mineral directly is an old idea, but one that fell out of favor in the 1980s and 1990s.^18^,^19^,^20^,^21^ Recent observations and the new appreciation of the functional versatility of osteocytes in regulating bone and mineral metabolism have shined a spotlight on this idea once again. The goal of this review is to consider the old and new evidence for ‘osteocytic osteolysis', the concept that osteocytes can, themselves, remove and replace bone.

## Can osteocytes resorb bone?

The initial suggestions that osteocytes could remove mineralized bone from around their lacunae are ascribed to Rigal and Vignal and von Recklinghausen.^22^,^23^ However, Baud^24^ has been credited with the first detailed morphological studies demonstrating periosteocytic osteolysis. Using electron microscopy Baud found evidence of enlarged lacunae with irregular borders, rough walls and varying degrees of perilacunar demineralization surrounding mature osteocytes. The numbers of these enlarged lacunae could be increased by the administration of parathyroid hormone (PTH) and decreased by the administration of calcitonin. Similarly, Krempien *et al*.^25^ reported that immobilization of rats by spinal cord or nerve transection resulted in an increase in the number of enlarged osteocyte lacunae with destruction of the lacunar wall, fragmentation of collagen fibrils and loss of mineral crystals upon ultrastructural examination. These findings were prevented by thyroparathyroidectomy. In an additional study, the same group demonstrated that the acute or chronic administration of PTH to rats resulted in widening of the lacunae with lysis of the lacunar wall as well as an increase in the rough endoplasmic reticulum, the number of lysosomes and cytoskeletal changes that suggested to the authors that the osteocytes had become ‘activated'.^26^ Thus, from the very beginning, it was thought that, directly or indirectly, PTH could stimulate osteocytes to remove perilacunar mineral and collagen. A series of subsequent studies using histological and microradiographic techniques supported the idea that the administration of PTH resulted in the enlargement of osteocyte lacunae.^18^,^27^,^28^ In one such study, 4-week continuous administration of PTH to rats using osmotic mini pumps resulted in the enlargement of osteocyte lacunae in cortical bone based on histology and contact microradiography.^28^ In addition, PTH treatment induced the expression of acid phosphatase in a subset of osteocytes.^28^ Enlarged osteocyte lacunae have also been noted in histomorphometry studies of human patients with primary hyperparathyroidism.^29^ In summary, multiple older studies suggested that PTH signaling could induce osteocytic osteolysis.

Several other conditions have been associated with osteocyte bone remodeling. Perhaps related to the effects of elevated PTH, restricting calcium intake has been shown to increase osteocyte lacunar size and enlarged osteocyte lacunae have been reported in patients with renal osteodystrophy as well, another condition associated with secondary hyperparathyroidism.^18^,^30^,^31^ Studies of the effects of space flight on rats and monkeys have suggested that microgravity is associated with osteocytic osteolysis.^32^,^33^ The enlargement of osteocyte lacunae has been reported in bone near skeletal metastases, perhaps reflecting the local production of PTH-related protein (PTHrP) by tumors.^34^ Osteocytic osteolysis has been reported in hibernating bats, squirrels and hamsters.^35^,^36^,^37^ Snakes appear to rely on osteocytic bone resorption during winter months and during reproductive cycles.^38^ Finally, enlargement of osteocyte lacunae has been demonstrated in lactating bats and mice.^19^,^35^ The majority of these observations rely on purely histological and radiographic associations but, nonetheless, there is a considerable body of evidence to suggest that under conditions associated with increased calcium demand, osteocytes can mobilize perilacunar mineral stores. Furthermore, this process appears to be triggered by PTH.

Not all studies have found evidence for enlarged osteocyte lacunae. Weisbrode *et al*.^39^ did not find conclusive evidence for osteocytic osteolysis in thyroparathyroidectomized rats placed on a low-calcium diet and given PTH. Rasmussen^40^ studied lactating rats maintained on a low-calcium diet and also did not find evidence for osteocytic osteolysis. Finally, Sissons *et al*.^41^ did not find evidence for periosteocytic osteolysis in virgin rats fed a low-calcium diet, although they noted that ostocytes newly formed after the commencement of calcium restriction had slightly larger lacunae. This later point, that newly formed osteocytes have larger lacunae, has frequently been cited to question whether the observations of enlarged osteocyte lacunae are simply a consequence of an increased number of young osteocytes in states of increased bone turnover.^21^ However, the majority of the previously cited reports acknowledged this issue and claimed to see enlargement of the lacunae surrounding mature osteocytes, although it is not clear how the investigators controlled for the age of the osteocytes in their samples.

## Can osteocytes form bone?

There have been fewer studies examining the bone-forming activity of osteocytes. Based primarily on the ultrastructural characteristics of the osteocytes and the perilacunar matrix, Jande and Belanger^42^ proposed that osteocytes could form new bone and gradually decrease the size of the lacunae. However, this was initially described to be a function of recently formed osteocytes. Baylink and Wergedal^43^ were the first to show direct evidence for periosteocytic mineralization. They treated rats with daily tetracycline for 17 days and found labeling within the osteocyte lacunar and canalicular spaces, suggesting the formation of new mineralized tissue. Again, this process seemed to be associated primarily with young osteocytes. Additional support for osteocyte matrix deposition came from a study that documented osteopontin deposition within osteocyte lacunae using immune-electron microscopy.^44^ These investigators found multiple concentric rings of osteopontin around some osteocytes, suggesting perhaps that these cells had undergone a cyclical process of bone formation. Finally, Zambonin Zallone *et al*.^45^,^46^ have studied osteocyte bone deposition using laying hens that had been deprived of dietary calcium and then repleted. During the calcium repletion phase, there is avid bone formation. Using ^3^H-proline pulses, histology and tetracycline labeling, these investigators were able to document that up to 20% of osteocyte lacunae were sites of active matrix and mineral deposition.^45^,^46^ However, none of these previous studies have documented that bone formation by osteocytes is preceded by osteolysis.

## Osteocytic osteolysis refuted

Despite the evidence cited above, there is much skepticism over the concept that osteocytes can actively remodel perilacunar bone. This stems from several arguments that led this concept to become discredited in the late 1970s. One of the issues at the time was that osteocytic osteolysis had become coupled with another concept called ‘bone flow'. This theory held that bone formation occurred primarily at bone surfaces, but that bone resorption occurred primarily in the interior as a result of osteocytic osteolysis.^18^,^47^ The idea was that bone tissue flowed somewhat like a glacier from the surface toward the osteocytes. The osteocytic osteolysis/bone flow theory attempted to explain the relative paucity of osteoclasts on the bone surface and assumed that there were insufficient numbers of osteoclasts to explain normal bone turnover and calcium homeostasis.^18^,^47^ In a paper published in 1977, Parfitt^21^ argued persuasively that bone flow did not occur and that the resorbing capacity of osteoclasts was, indeed, sufficient to account for physiological bone remodeling and skeletal mineral release. Furthermore, he argued that the observations purporting to show enlargement of osteocyte lacuna were likely to be artifacts of tissue preparation and the difficulty of two-dimensional histological and radiographic techniques to account for osteocyte orientation and variablility in the stage of osteocyte maturation.^21^ The concept that osteocytes could resorb bone was further called into question when isolated osteocytes were found to be incapable of resorbing dentin in culture.^48^ Given that the concept of bone flow was debunked and given the general acceptance that osteoclasts were the principal bone-resorbing cell, interest and research in the potential remodeling activity of osteocytes fell off greatly. It became widely accepted that even if osteocytic bone resorption did occur, this process was not likely to be important.

## Reversible osteocytic perilacunar and canalicular remodeling during lactation

Lactation requires a great deal of calcium, as milk must supply all the mineral resources required for rapid skeletal growth in the neonatal period.^49^,^50^,^51^,^52^ Similar demands for calcium occur in lower vertebrates during egg production and shell calcification.^51^ In both lower vertebrates and in mammals, the maternal skeleton is an important source of mineral for reproduction; bone mineral is mobilized for egg production and milk production. In nursing women, bone mineral density declines by 5–8% over the course of 6 months of lactation and lactating rodents lose up to one-third of their skeletal mass after 3 weeks of suckling.^49^,^50^,^51^,^52^ This process of rapid bone loss is associated with increased bone turnover and increased numbers of surface osteoclasts and bone resorption. Furthermore, this process is triggered in part by the secretion of PTHrP from the breast.^53^,^54^,^55^ PTHrP interacts with the same type 1 PTH/PTHrP receptor (PTHR1) in bone as does PTH. Given that lactation is associated with rapid bone loss, pronounced negative calcium balance and the activation of skeletal PTHR1 signaling, it seemed to us that it would be a likely situation in which physiological osteocytic bone remodeling might occur. Therefore, in collaboration with the Bonewald and Pajevich laboratories, we examined whether osteocytic osteolysis participated in the regulation of bone and calcium metabolism in lactating mice.

**Figure 1:**
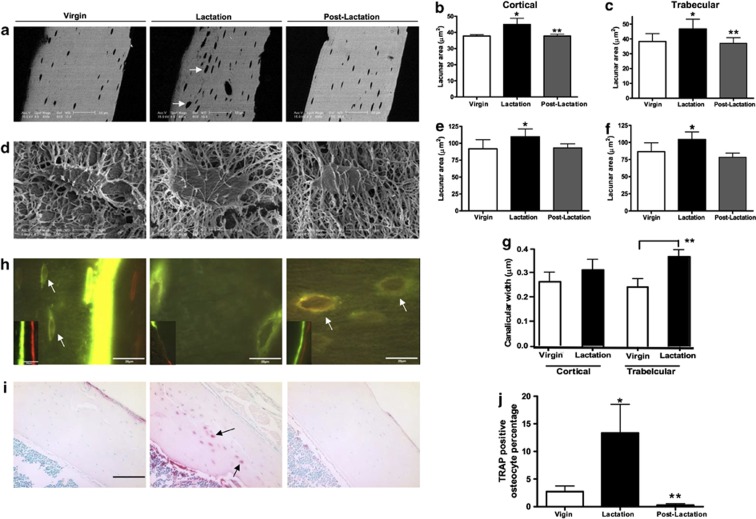
Lactation induces osteocytic lacunar and canalicular enlargement during lactation that returns to virgin levels post lactation. (**a**) Backscatter electron microscopy images showing reversible enlargement of osteocyte lacunae during lactation. (**b**, **c**) Quantification of osteocyte lacunar area in virgin, lactating and post-lactation mice. (**d**) Typical acid-etched, resin-casted scanning electron microscopy (SEM) images used to assess osteocyte lacunar size. (**e**, **f**) Quantification of lacunar size using acid-etched, resin-casted SEM images of osteocyte lacunae from virgin, lactating and post-lactation mice. Note the reversible increase in lacunar size during lactation using both backscatter EM and acid-etched SEM. (**g**) Quantification of canalicular diameter from acid-etched, resin-casted SEM images from virgin and lactating mice. (**h**) Double fluorochrome labeling of bone from virgin, lactating and post-lactation mice. Distinct double labels can be found at the surface (insets) of virgin and post-lactating mice. Only an intermittent single label is seen at the surface of lactating bone given the rapid turnover. In virgin mice, some label was taken up in osteocyte lacunae near the mineralization front (white arrows, left panel). However, osteocyte lacunae distant from the mineralization front were labeled with both fluorochromes in post-lactating mice, suggesting active mineralization around osteocytes in the period of recovery from lactation. (**i**) Tartrate resistant acid phosphatase staining showed more TRAP-positive osteocytes during lactation (black arrows). (**j**) Osteocytes with TRAP activity from the lactating mice (13.4±5.2%* TRAP+ osteocytes) was significantly increased compared with the virgin (2.7±1.8%) and day 7 post-lactation (0.3±0.5%**) animals. *P<0.05; **P<0.01. Reproduced with permission from Qing *et al*.^27^

**Figure 2:**
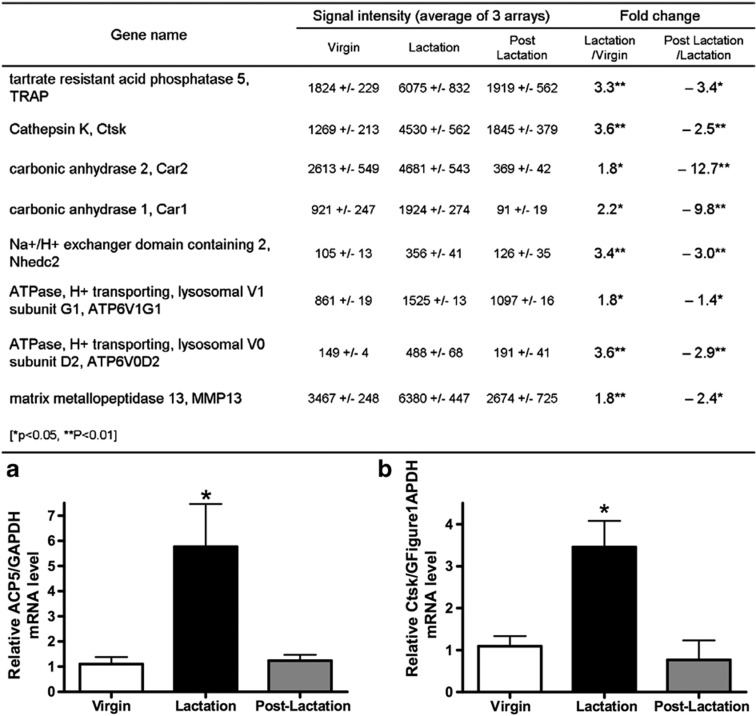
Expression of several osteoclast-specific genes is increased in osteocytes during lactation. Table displaying genes potentially involved in osteoclast-like bone resorption whose expression was reversibly elevated in osteocytes from lactating as compared with virgin or post-lactation mice. (**a**) Validation of microarray using quantitative (q)PCR examining expression of ACP5 (tartrate-resistant acid phosphatase (TRAP)) mRNA from osteocytes from lactating vs virgin and post-lactation mice. (**b**) Validation of microarray using qPCR examining expression of Ctsk (cathepsin K) mRNA from osteocytes from lactating vs virgin and post-lactation mice. *P<0.05; **P<0.01. Reproduced with permission from Qing *et al*.^27^

**Figure 3:**
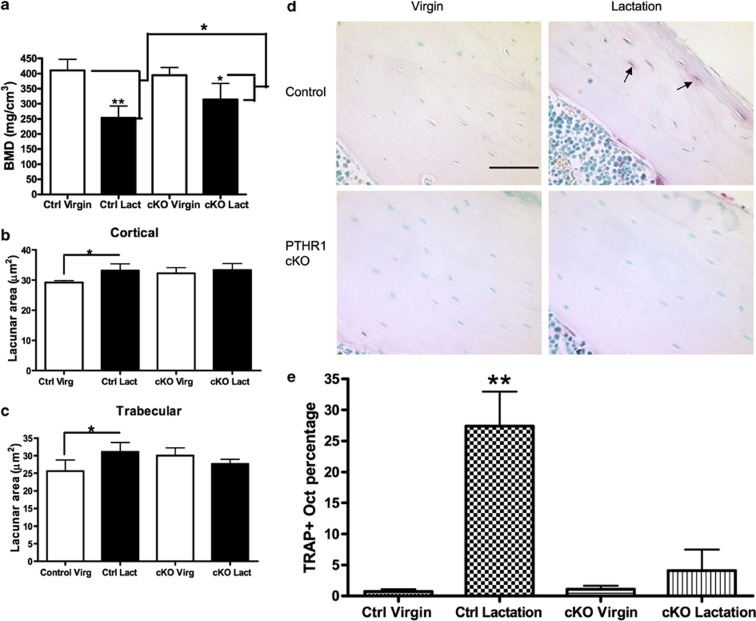
Osteocyte remodeling during lactation is blocked by osteocyte-specific disruption of the *PTHR1* gene (PTHR1 CKO). (**a**) Bone mineral density (BMD) as measured by micro-computed tomography in virgin and lactating PTHR1 CKO mice. Bone loss during lactation is attenuated by disruption of the PTHR1 in osteocytes. Measurement of osteocyte lacunar area in cortical bone (**b**) and trabecular bone (**c**) of virgin and lactating control and PTHR1 CKO mice. Loss of the PTHR1 in osteocytes prevents the increase in lacunar size that occurs during lactation in controls. (**d**) Tartrate-resistant acid phosphatase (TRAP) staining in osteocytes in virgin and lactating control and PTHR1 CKO mice. (**e**) Quantification of the numbers of TRAP-positive osteocytes in virgin and lactating, control and PTHR1 CKO mice. In controls, many osteocytes become TRAP positive during lactation, but activation of TRAP expression is prevented in lactating PTHR1 CKO mice. *P<0.05; **P<0.01. Reproduced with permission from Qing *et al*.^27^

We used backscatter electron microscopy and acid-etched scanning electron microscopy to examine the size of osteocyte lacunae in virgin, lactating and post-lactation recovered mice.^56^ As shown in Figure 1, the size of osteocyte lacunae in both trabecular and cortical bone increased in lactating mice as compared with virgin mice, but 7 days after the end of lactation, the size of the lacunae had decreased back to the baseline of virgin mice. Furthermore, we detected evidence for dual fluorochrome labeling of the osteocyte lacunae during the period of recovery from lactation, demonstrating tetracycline labeling in these sites (Figure 1h). These changes were not simply a nonspecific response to increased bone turnover as they were not present in the calvarium of lactating mice and they did not occur in the setting of tail suspension, even though this maneuver was associated with a degree of bone loss equivalent to that observed during lactation. In a related study, we used a combination of micro-computed tomography-based analyses including individual trabecular segmentation and digital topological analysis-based tissue mineral density measurements and found that there was a reversible decrease in trabecular central tissue mineralization during lactation, a finding consistent with removal of bone mineral by osteocytes.^57^ Histochemical staining and gene array studies demonstrated that lactation is associated with the reversible activation in osteocytes of genes typically associated with osteoclastic bone resorption. These include tartrate-resistant acid phosphatase, cathepsin K, carbonic anhydrase, matrix metallopeptidase 13 and several subunits of the H^+^-ATPase (Figure 2). Interestingly, a recent study from Tang *et al*.^58^ confirmed that matrix metallopeptidase 13 contributes to osteocyte perilacunar and canalicular remodeling in cortical bone during lactation. We also found that infusing virgin mice with PTHrP 1–36 for 11 days induced an increase in the size of the osteocyte lacuna and induced the expression of tartrate-resistant acid phosphatase in osteocytes.^53^,^56^ Finally, conditional disruption of the *PTHR1* gene in osteocytes using the DMP1-Cre transgenic mouse completely blocked the increase in lacunar size and the induction of tartrate-resistant acid phosphatase activity normally observed during lactation (Figure 3).^56^ Therefore, we concluded that lactation is associated with reversible periosteocytic bone remodeling and that PTHrP-PTHR1 signaling in osteocytes activates a bone-resorbtion program during lactation that uses some of the same acid-protease mediators that are used in surface bone resorption by osteoclasts.

## Perspective and conclusions

As summarized previously, there have been many past observations of ostoecytic osteolysis. However, the concept fell out of favor primarily due to the fact that its proponents thought it to be the dominant form of bone resorption, which proved not to be the case. Our recent findings suggest that reversible, PTHR1-mediated perilacunar and canalicular remodeling is a physiological response to lactation. Our observations could be consistent with either perilacunar/canalicular demineralization or with osteocyte-mediated removal of both mineral and matrix. Further experiments will be required to clarify this point. Nevertheless, our studies do demonstrate that osteocytes can mobilize and replace bone mineral in response to the cyclical demands for calcium that occur during reproduction. Perhaps then, it is time to reconsider ‘osteocytic osteolysis' and consider how it may fit into the regulation of bone and mineral metabolism more broadly. There are many questions about this process that need clarification. The following is not a comprehensive list, but includes some obvious starting points for further research.

1. How quantitatively important is osteocytic remodeling to the bone loss that occurs in lactation? Our data demonstrate that reversible perilacunar and canalicular remodeling occurs during reproductive cycles, but it does not shed light on how much this process contributes to the mobilization of skeletal calcium stores for milk production. The vast surface area of the bone–osteocyte interface makes it an attractive site for the exchange of mineral between the skeleton and systemic circulation during lactation. Overall bone loss during lactation was reduced in the DMP1-Cre;PTHR1^lox/lox^ mice, but, at present, it is not clear whether this is due to a reduction in surface bone activity, the loss of osteocyte remodeling or both.
2. Does osteocytic remodeling contribute to the regulation of bone mass or mineral homeostasis in other settings? It remains to be seen whether the ostecyte is an important contributor to routine mineral homeostasis or whether it acts as a back-up system for times of stress such as reproduction.
3. How does PTH signaling induce cells in the osteoblast lineage to express a gene program that encodes machinery of bone resorption that is typically characteristic of osteoclasts? How closely do the molecular mechanisms used by osteocytes to remove bone mineral and/or matrix resemble those used by osteoclasts? Can PTH induce similar processes in other osteoblasts or in bone-lining cells? While the changes in gene expression listed in Figure 2 were generated from osteocyte-enriched bone and not by a pure osteocyte population, they provide an initial roadmap that can direct efforts to begin to answer these questions.
4. Does osteocytic remodeling affect the activity of surface osteoclasts and osteoblasts? Osteocytes have been shown to regulate bone turnover, and osteocytic osteolysis has been noted in the vicinity of active bone resorption by osteoclasts. Recently, it has become appreciated that osteocytes are an important source of RANKL production in bone that is particularly critical for maintaining osteoclast activity in the bone remodeling cycle.^59^,^60^,^61^ Thus, it is possible that during lactation, PTHrP-PTHR1 signaling stimulates osteocytes to both remodel their immediate environment and to activate osteoclast-mediated surface bone resorption by upregulating RANKL production. Likewise, osteocytes have been shown to regulate osteoblast activity by regulating sclerostin production.^1^,^2^ Perhaps the remodeling activity of osteocytes during lactation might influence the activity of surface osteoblasts and osteoclasts by altering the production of RANKL, sclerostin or other cytokines.
5. Does osteocytic remodeling alter mechanical–biochemical coupling in bone? It has long been established that the osteocyte lacunar-canalicular network is important in mediating the effects of mechanical force on the skeleton. It is possible that changes in the size of the lacunae and canaliculi or the material properties of the surrounding matrix might modulate how these cells respond to loading.

In closing, it would appear that osteocytic osteolysis occurs during lactation and that, at least in mice, osteocytes can remove and then replace bone mineral surrounding their lacunar-canalicular network. Given the advances in our understanding of the versatile functions of osteocytes in regulating bone and mineral metabolism, it is probably time to reconsider whether osteocyte perilacunar and canalicular remodeling may be involved in skeletal physiology more generally.
